# Driving with a neurodegenerative disorder: an overview of the current literature

**DOI:** 10.1007/s00415-017-8489-9

**Published:** 2017-04-19

**Authors:** Milou Jacobs, Ellen P. Hart, Raymund A. C. Roos

**Affiliations:** 1 Department of Neurology, Leiden University Medical Center, Albinusdreef 2, 2333 ZA Leiden, The Netherlands; 2 Center for Human Drug Research, Leiden, The Netherlands

**Keywords:** Driving, Neurodegenerative disorders, Movement disorders, Cognitive assessment, Review

## Abstract

Driving is important for employment, social activities, and for the feeling of independence. The decision to cease driving affects the quality of life and has been associated with reduced mobility, social isolation, and sadness. Patients with neurodegenerative disorders can experience difficulties while driving due to their cognitive, motor, and behavioral impairments. The aim of this review is to summarize the available literature on changes in driving competence and behavior in patients with neurodegenerative disorders, with a particular focus on Huntington’s (HD), Parkinson’s (PD), and Alzheimer’s disease (AD). A systematic literature search was conducted in the PubMed/Medline database. Studies using on-road or simulated driving assessments were examined in this review. In addition, studies investigating the association between cognitive functioning and driving were included. The review identified 70 studies. Only a few publications were available on HD (*n* = 7) compared to PD (*n* = 32) and AD (*n* = 31). This review revealed that driving is impaired in patients with neurodegenerative disorders on all levels of driving competence. The errors most commonly committed were on the tactical level including lane maintenance and lane changing. Deficits in executive functioning, attention, and visuospatial abilities can partially predict driving competence, and the performance on neuropsychological tests might be useful when discussing potential driving cessation. Currently, there is no gold standard to assess driving ability using clinical measures such as neuropsychological assessments, so more studies are necessary to detect valid screening tools and develop useful and reliable evidence-based guidelines.

## Introduction

Progressive neurodegenerative diseases can result in a loss of motor and cognitive functioning, which interfere with daily activities such as the ability to drive a car [1]. Many individuals rely on their car for employment, social activities, and independency [2–4]. Therefore, the decision to cease driving affects the quality of life. Driving cessation has been associated with negative outcomes such as social isolation, reduced mobility, and sadness [5]. A difficult question that clinicians face in everyday practice is when to advise patients with early disease to abstain from driving. In most European countries, neurologists evaluate driving competence in patients with neurodegenerative disorders, based on their clinical examination [6]. Depending on the outcome of this evaluation, patients can be advised to contact an official national driving evaluation center. However, the evaluations of neurologists are often an overestimation of the actual driving capacities and inconsistent with on-road performances [3]. In the Netherlands, a neurologist has to evaluate if a patient should perform a formal driving test [7]. However, the decision to inform the national driving evaluation center relies on the self-report of patients. If a patient passes the formal driving test, the driver license can be renewed with a maximum of 5 years. Within this 5-year period, patients have no obligation to perform a retest. This can potentially be unsafe with the progressive character of neurodegenerative diseases, especially since changes in cognitive and daily functioning can already occur within 5 years [8, 9].

The aim of this review is to provide an overview of the available literature on changes in driving competence in patients with neurodegenerative disorders and to identify potential gaps in the literature that should be further investigated, with particular interest for Huntington’s disease (HD), Parkinson’s disease (PD), and Alzheimer’s disease (AD). We focused on these neurodegenerative disorders, since they are comparable in cognitive, psychiatric, and motor symptoms. A comprehensive review incorporating all three diseases has not been published before. Furthermore, we evaluate if specific cognitive tests have been identified that are predictive of driving ability and if these tests can be implemented in the clinical practice. Since simulators are increasingly being used in driving research and might be a proper screening tool to assess driving in patients with neurodegenerative diseases, we also included available literature on driving simulators.

## Methods

An electronic database search in PubMed/MEDLINE was performed to identify the available literature. The last database search was performed on 27th October 2016. The following search terms were used individually and in combination: “driving” “driving ability” “neurodegeneration”, “Huntington’s disease”, “Huntington”, Parkinson’s disease”, “Parkinson”, “Alzheimer’s disease”, “Alzheimer”, “dementia”, “cognition”, “cognitive functioning”, and “simulator”. In addition, references and reviews were checked in search of relevant studies. In the initial search, only papers written in English were considered and selected for further review. Only original articles and full communications were included (e.g., no letters to editors, editorial comments, or reviews). Articles were deemed relevant if they directly investigated driving-related issues using formal driving assessments (i.e., on-road or simulator) in diagnosed patients with HD, PD, or AD.

## Results

### Search results

The database search yielded 240 articles that were selected for further review based on title. The abstract of each article was reviewed and the inclusion/exclusion criteria were checked. From these 240 articles, 70 studies met the inclusion criteria of the current review (7 HD, 32 PD, and 31 AD studies). The majority of the studies described on-road driving performances (*n* = 45), 21 studies involved driving simulation, and 51 articles investigated the relationship between cognitive performances and driving outcomes. A summary of the included literature and the methods that were used is given per group in Tables 1, 2, and 3. When applicable, we will use the driving model of Michon et al. [10]. According to this model, driving errors can be sorted in three categories: (a) strategic errors that occur before actual driving, such as route planning; (b) tactical errors consisting of errors in speed adaptations, changing lanes, and keeping distance; (c) operational errors such as incorrect responses to changing driving environments and vehicle control [11, 12]. An overview of the committed driving errors by patient group per category is given in Table 4.

**Table 1:** Study details of included studies on Huntington’s disease

Authors (year)	Number of participants (*n*) HD/controls (C)	Age (years) mean ± SD HD/controls (C)	Driving assessment	Cognitive/motor assessments	Main findings
Beglinger et al. (2010)	265 HD at-risk/no C	44.5 ± 12.4	Questionnaire	UHDRS-TMS, TFC, FAS, Stroop, Verbal fluency, SDMT	33.5% (86/265) reported inability to drive safely Motor functioning and Stroop test were significantly associated with driving safety item of a questionnaire
Beglinger et al. (2012)	74 HD/no C	48.2 ± 12.3	Driving status determined by chart review	UHDRS-TMS, TFC, Verbal fluency, SDMT, Stroop, RBANS, TMT, WAIS-III information, letter-number sequencing, similarities	Motor, cognitive, and functional decline were associated with driving Cognitive impairment was the most strongest risk factor for driving cessation
Devos et al. (2012)	30 HD/30 C	HD: 50.2 ± 12.4/C: 50.3 ± 12.6	On-road and simulator	UHDRS-TMS, TFC, Verbal fluency, Stroop, SDMT, TMT, MMSE	50% of HD patients failed the on-road evaluation (controls did not perform on-road assessment) Pass/fail scores of the on-road assessment were best predicted by a combination of the SDMT, Stroop word, and TMT-B tasks (sensitivity/specificity = 87%)
Devos et al. (2014)	30 HD/30 C	HD: 50.2 ± 12.4/C: 50.3 ± 12.6	On-road	UHDRS-TMS, Verbal fluency, Stroop, SDMT, TMT, UFOV, Visual scanning, Divided attention	47% of the HD patients (14/30) failed the on-road evaluation versus none of the controls HD patients scored worse than controls on all items of the road test Selective attention was the only predictor that correlated with all clusters of the on-road score
Hennig et al. (2014)	52 HD/no C	HD (referred to DMV): 47.3 ± 11.0/HD (not referred to DMV): 45.0 ± 12.3	On-road	RBANS coding, TMT part B, Stroop, CalCAP sequential reaction time	31/52 HD patients were referred to DMV for a driving evaluation Association between neuropsychological assessments and driving competence
Rebok et al. (1995)	73 HD 29 HD/16 C for simulator study	HD: 43.8 ± 11.9 Not reported for simulator study	Simulator	MMSE, WAIS-R vocabulary, block design, VMI, FAS, BTA, HVLT, TMT, Stroop, WCST, WMS-R logical memory, visual reproduction, Motor-free VPT, Spatial recognition test, Reaction time task	53/73 (72%) continued driving after disease onset HD patients had higher error rates on the driving simulator outcomes and lower cognitive scores compared to healthy individuals
Williams et al. (2011)	16 HD/no C	65.6 ± 10.0	Semi-structured interview	–	Driving was the most common endorsed item (11/16) HD patients reported lower reaction times, difficulties multi-tasking, and concerns about safety

*C* controls, *CalCAP* California Computerized Assessment Package, *DMV* department of motor vehicles, *FAS* Oral Word Association Test, *HD* Huntington’s disease, *HVLT* Hopkins Verbal Learning Test; *MMSE* Mini-Mental State Examination, *RBANS* Repeatable Battery for the Assessment of Neuropsychological Status, *SDMT* Symbol Digit Modalities Test, *TFC* total functional capacity, *TMT* Trail Making Test, *UFOV* useful field of view, *UHDRS-TMS* Unified Huntington’s Disease Rating Scale-Total Motor Score, *VMI* visual motor integration, *VPT* Visual Perception Test, *WAIS-III* Wechsler Adult Intelligence Scale-III, *WAIS-R* Wechsler Adult Intelligence Scale-Revised, *WCST* Wisconsin Card Sorting Test, *WMS-R* Wechsler Memory Scale-Revised, *WMS-III* Wechsler Memory Scale-Third edition

**Table 2:** Study details of included studies on Parkinson’s disease

Authors (year)	Number of participants (*n*) PD/controls (C)	Age (years) mean ± SD PD/controls (C)	Driving assessment	Cognitive/motor assessments	Main findings
Amick et al. (2007)	25 PD/no C	PD (safe): 62.9 ± 8.9 PD (marginal): 66.1 ± 6.5	On-road	UPDRS motor, Contrast sensitivity, ROCF, TMT, UFOV, Backwards visual masking, FACT, Pelli–Robson	11/25 (44%) PD patients had marginal or unsafe rating on the road test Composite measure of executive functioning and visuospatial abilities correctly classified 71.4% of safe drivers and 72.7% of marginal unsafe drivers
Classen et al. (2009)	19 PD/104 C	PD: 74.8 ± 6.1/C: 75.4 ± 6.4	On-road	UPDRS, UFOV, MMSE, TMT part B, Contrast sensitivity tests	8/19 (42%) PD patients failed the on-road assessment versus 21.2% controls (22/104) UFOV scores showed strongest correlation with on-road performance UFOV risk index: cutoff = 3, sensitivity = 87%, specificity = 82%; UFOV divided attention: cutoff = 223 ms, sensitivity = 87.5%, specificity = 81.8%; UFOV selected attention: cutoff = 273 ms, sensitivity = 75%, specificity = 72.7%
Classen et al. (2011)	41 PD/41 C	PD: 73.1 ± 6.0/C: 73.0 ± 5.2	On-road	UPDRS, Rapid pace walk, MMSE, UFOV, Contrast sensitivity tests	56% of PD patients failed the on-road assessment versus 12.2% of controls Model with UFOV divided attention and Rapid pace walk accurately classified 80.5% of PD subjects in pass/fail category (sensitivity = 82.6%, specificity = 77.8%)
Classen et al. (2014)	101 PD/138 C	PD: 69.4 ± 7.4/C: 71.8 ± 5.1	On-road	–	41% of PD patients failed the on-road assessment versus 9% of controls Errors in visual scanning, signaling, vehicle positioning, and speed regulation were most predictive of overall pass/fail scores
Classen et al. (2015)	99 PD/no C	Not reported (range 35–89)	On-road	UPDRS motor, TMT part B, FNT, rapid pace walk, contrast sensitivity	Poorer performance on the clinical variables was associated with more driving errors. Contrast sensitivity, TMT part B, and FNT were predictors of on-road performance
Cordell et al. (2008)	53 PD/129 C	PD: 69.3 ± 8.3/C: 72.9 ± 7.1	On-road	–	Control group performed better on all driving tasks Most common errors by PD patients were failing to check blind spot, unsteady car speed, and inappropriate signaling at roundabouts
Crizzle et al. (2013)	27 PD/20 C	PD: 71.6 ± 6.6/C: 70.6 ± 7.9	On-road	UPDRS motor, Pelli–Robson, MoCA	PD patients had lower reaction times and worse cognitive scores compared to controls. Reaction time was negatively associated with night driving
Crizzle et al. (2013)	27 PD/20 C	PD: 71.6 ± 6.6/C: 70.6 ± 7.9	On-road	–	PD patients had a more restricted driving pattern compared to controls with less driving at night and during bad weather
Crizzle et al. (2013)	55 PD/no C	71.0 ± 7.0	On-road	UPDRS motor, Rapid pace walk, Modified Hoehn and Yahr	28/55 (51%) of PD patients failed the road test Combined scores of Rapid pace walk and Modified Hoehn and Yahr best predictor of safe driving MHY score of ≥2.5: sensitivity = 61%, specificity = 78%; RPW score of ≥6.22: sensitivity = 68%, specificity = 89%
Devos et al. (2007)	40 PD/40 C	PD: 61.6 ± 9.4/C: 62.8 ± 7.6	On-road and simulator	UPDRS motor, UPDRS ADL, CDR, Pelli–Robson, ROCF, UFOV, Visual scanning tests, Attention tasks	11/40 (27.5%) of PD patients failed the on-road test (controls did not perform on-road assessment) PD patients performed worse on the driving simulator score and made more traffic offences compared to controls Adding a driving simulator to screening battery increased accurate classification from 90 to 97.5% (sensitivity = 91%, specificity = 100%)
Devos et al. (2013)	60 PD/no C	PD (pass): 62.7 ± 9.7/PD (fail): 71.1 ± 7.1	On-road and simulator	UPDRS motor, Pelli–Robson, CDR	40% of PD patients failed the on-road assessment Predictive model accurately classified 46 drivers in pass/fail category (sensitivity = 96%, specificity = 94%)
Devos et al. (2013)	104 PD/no C	66.0 ± not reported	On-road	Binocular acuity, kinetic vision, Pelli–Robson, UPDRS motor, UFOV, ROCF, Attention tasks, Visual scanning tests	35% of PD patients failed the on-road assessment The fail group performed worse on all other clinical tasks compared to pass group
Heikkilä et al. (1998)	20 PD/20 C	PD: 59.0 ± 11.0/C: 55.0 ± 6.0	On-road	Visual memory, Perception, Vigilance, Choice reactions, Information processing	PD patients had most difficulties driving in an urban area and committed more errors than controls Neurologist overestimated driving ability of PD patients
Madeley et al. (1990)	10 PD/10 C	PD: 54.6 ± not reported/C: 55.9 ± not reported	Simulator	–	Driving reaction time and steering accuracy were impaired in the PD patients
Radford et al. (2004)	51 PD/no C	64.4 ± 9.1	On-road	Webster’s rating scale, UPDRS motor, SDSA, AMIPB, Stroop, PASAT, Tapping task	6/49 (12%) PD patients were classified as unsafe drivers Unsafe drivers drove worse on roundabouts and had poorer road positioning. No differences in cognitive performance between safe and unsafe drivers with PD
Ranchet et al. (2011)	25 PD/25 C	PD: 65.4 ± 5.2/C: 66.7 ± 4.4	Simulator	UPDRS motor, MMSE, Stroop, TMT, BVRT, Digit span, PMT, N-back, Mental flexibility, Three tasks during driving simulator assessment	Updating information was impaired in PD patients compared to controls TMT was the best predictor of driving simulator outcome (explained 40.7% of variance on simulator test)
Ranchet et al. (2013)	19 PD/21 C	PD: 66.1 ± 5.1/C: 69.1 ± 3.9	On-road	UPDRS motor, MMSE, Stroop, TMT, BVRT, Digit span, PMT, N-back, Mental flexibility	Driving performance was poorer in PD patients compared to controls Combination of cognitive measures discriminated between at-risk and safe drivers (sensitivity = 93.8%, specificity = 85.7%)
Ranchet et al. (2016)	25 PD (16 at follow-up)/25 C (21 at follow-up)	PD: 65.4 ± 5.2/C: 66.7 ± 4.4	Simulator	UPDRS motor, MMSE, Stroop test, TMT, BVRT, Digit span, PMT, N-back, Mental flexibility, Three tasks during driving simulator assessment	At follow-up, PD patients performed worse compared to controls on updating information during the simulator
Scally et al. (2011)	19 PD/19 C	PD: 68.7 ± 6.7/C: 68.05 ± 7.2	Simulator	UPDRS motor, MMSE, WMS-III digit span, WMS-III mental control, TMT	PD patients showed delayed initiation in braking response Slower psychomotor speed and poorer attention was associated with earlier braking in the PD group
Singh et al. (2007)	154 PD/no C	67.6 ± not reported	On-road	–	50/154 (32.5%) of PD patients were unsuitable to drive
Stolwyk et al. (2005)	18 PD/18 C	PD: 67.6 ± 6.5/C: 67.1 ± 6.5	Simulator	UPDRS motor, MMSE	PD patients relied more on external than internal cues to regulate driving compared to controls
Stolwyk et al. (2006)	18 PD/18 C	PD: 67.6 ± 6.5/C: 67.1 ± 6.5	Simulator	UPDRS motor, MMSE	PD patients drove more cautious than controls
Stolwyk et al. (2006)	18 PD/18 C	PD: 67.6 ± 6.5/C: 67.1 ± 6.5	Simulator	UPDRS motor, MMSE, Up-and-Go test, TMT, SDMT, Reaction time tests, Brixton test, WAIS-III picture completion, WAIS-III digit span, WAIS-III block design, JLO	Correlations between specific neuropsychological tests and driving outcome variables TMT-B, Brixton test, and Block design correlated with tactical errors; SDMT, Picture completion, and JLO correlated with operational errors
Uc et al. (2006)	79 PD/151 C	PD: 65.9 ± 8.6/C: 65.3 ± 11.5	On-road	UFOV, Pelli–Robson, Visual acuity, UPDRS, JLO, MMSE, CFT, BVRT, TMT, AVLT, COWA, Blocks, Structure from Motion test	PD patients committed more safety errors and identified fewer traffic signs and landmarks compared to controls Specific neuropsychological tests (TMT, UFOV, CFT) correlated with driving outcome
Uc et al. (2006)	71 PD/147 C	PD: 66.0 ± 8.6/C: not reported	On-road	UFOV, Pelli–Robson, Visual acuity, UPDRS, JLO, MMSE, CFT, BVRT, TMT, AVLT, COWA, WAIS-R block design, PASAT	Driving safety decreased in PD group during distraction Cognitive and motor functioning predicted effects of distraction in the PD group
Uc et al. (2007)	77 PD/152 C	PD: 65.9 ± 8.6/C: 65.3 ± 11.5	On-road	UFOV, Pelli–Robson, Visual acuity, UPDRS, JLO, MMSE, CFT, BVRT, TMT, AVLT, COWA, WAIS-R block design	PD patients made more incorrect turns, safety errors, and got lost more often than controls. Poor performance on CFT and UFOV was predictive of driving errors
Uc et al. (2009)	84 PD/182 C	PD: 67.3 ± 7.8/C: 67.6 ± 7.5	On-road	UFOV, Pelli–Robson, Visual acuity, UPDRS, JLO, MMSE, CFT, BVRT, TMT, AVLT, COWA, WAIS-R block design	PD patients committed more total safety errors compared to controls (41.6 versus 32.9); lane violations were the most common error Visual processing speed, attention, and visual acuity were predictive of total number of errors
Uc et al. (2009)	67 PD/51 C	PD: 66.2 ± 9.0/C: 64.0 ± 7.2	Simulator	UFOV, Pelli–Robson, Visual acuity, UPDRS, JLO, MMSE, CFT, BVRT, TMT, AVLT, COWA, WAIS-R block design	PD patients had higher SDLP and lane violations during fog conditions compared to controls
Vardaki et al. (2016)	10 PD/10 C	PD: 62.2 ± 8.4/C: 57.6 ± 5.1	Simulator	MMSE, FAB, SDMT, UFOV, HVLT-R, TMT, WMS letter-number sequencing, spatial span task, Spatial addition test, Driving scenes test	No differences between PD patients and controls in sign recall after driving PD patients performed worse on the neuropsychological tests compared to controls
Wood et al. (2005)	25 PD/21 C	PD: 63.7 ± 6.8/C: 65.2 ± 8.6	On-road	UPDRS motor	14/25 (56%) PD patients failed the on-road driving test versus 5/21 (24%) controls PD patients made more safety errors compared to controls (e.g., lane keeping, reversing, parking)
Worringham et al. (2006)	25 PD/21 C	PD: 63.7 ± 6.8/C: 65.2 ± 8.6	On-road	UPDRS motor, MMSE, UFOV, Visual acuity, Pelli–Robson, Motion sensitivity, SDMT, TMT, Stroop, Purdue Pegboard, Reaction time task	Motor performance (Purdue pegboard), contrast sensitivity (Pelli–Robson) and cognitive function (SDMT) predicted pass/fail category in PD group (sensitivity = 72.7%, specificity = 64.3%)
Zesiewicz et al. (2002)	39 PD/25 C	PD: 63.8 ± 11.5/C: 65.6 ± 10.3	Simulator	UPDRS motor	PD patients had more total collisions compared to controls Motor functioning was associated with total number of collisions

*ADL* activities of daily living, *AMIPB* adult memory and information processing battery, *AVLT* Auditory Verbal Learning Test, *BVRT* Benton Visual Retention Test, *C* controls, *CDR* Clinical Dementia Rating Scale, *CFT* Complex Figure Test, *COWA* Controlled Oral Word Association, *FAB* frontal assessment battery, *FACT* Functional Acuity Contrast Test, *FNT* Finger to Nose Test, *HVLT* Hopkins Verbal Learning Test, *JLO* Judgement of Line Orientation Test, *MMSE* Mini-Mental State Examination, *MoCA* Montreal Cognitive Assessment, *PASAT* Paced Auditory Serial Addition Task, *PD* Parkinson’s disease, *PMT* plus minus task, *ROCF* Rey–Osterrieth complex figure, *SDLP* standard deviation of lateral position, *SDMT* Symbol Digit Modalities Test, *SDSA* Stroke Drivers Screening Assessment, *TMT* Trail Making Test, *UFOV* useful field of view, *UPDRS* Unified Parkinson’s Disease Rating Scale, *WAIS-III* Wechsler Adult Intelligence Scale-III, *WAIS-R* Wechsler Adult Intelligence Scale-Revised, *WMS-R* Wechsler Memory Scale Revised, *WMS-III* Wechsler Memory Scale-Third edition

**Table 3:** Study details of included studies on Alzheimer’s disease

Authors (year)	Number of participants (*n)* AD/controls (C)	Age (years) mean ± SD AD/controls (C)	Driving assessment	Cognitive/motor assessments	MMSE mean ± SD for AD/controls (C)	Main findings
Barco et al. (2015)	60 AD/32 C	AD: 74.2 ± 8.5/C: 70.7 ± 8.1	On-road	AD8, SBT, Clock drawing, TMT, Maze test, UFOV, Visual closure test	–	62% of AD patients failed the on-road test versus 3% of controls AD patients who failed made more errors in driving straight and turning compared to pass group
Bhalla et al. (2007)	84 AD/44 C	AD (safe): 75.3 ± 7.2/AD (unsafe): 77.3 ± 5.7/C: 73.6 ± 9.1	On-road	–	–	19% of AD patients were classified as unsafe drivers versus none of the controls
Bieliauskas et al. (1998)	9 AD/9 C	AD: 70.4 ± 6.0/C: 71.7 ± 4.6	On-road	MMSE, Visual search test, Reaction time test, Figure-ground perception test, WCST, SILS	AD: 19.4 ± 3.1/C: 27.9 ± 1.5	AD patients made more total driving errors compared to controls Errors in turning were the most frequent
Bixby et al. (2015)	75 AD/no C	76.6 ± 6.3	On-road	–	–	Ratings by clinicians and spouses were poorly associated with driving performance. Ratings by adult children were most related to driving
Brown et al. (2005)	31 AD/24 C	AD: 76.9 ± 5.4/C: 72.0 ± 10.3	On-road	–	AD: 25.1 ± 3.6/C: 29.1 ± 1.2	AD patients performed worse compared to controls on the road test
Brown et al. (2005)	50 AD/25 C	AD (mild): 73.2 ± 8.3/AD (very mild): 77.1 ± 5.3/C: 72.4 ± 10.2	On-road	–	AD (mild): 21.5 ± 3.9/AD (very mild): 24.9 ± 3.6/C: 29.1 ± 1.2	AD patients had worse overall driving scores compared to controls 9/50 (18%) were classified unsafe by driving instructor versus none of the controls Prediction by physician was associated with driving test
Carr et al. (2011)	99 AD/no C	74.2 ± 9.0	On-road	AD8, Visual acuity, Pelli–Robson, SBT, Clock drawing, TMT, Digit span, UFOV, Visual perceptual test, SMT, Rapid pace walk, 9-hole peg test	–	65% of AD patients failed the on-road test Combination of clinical tests was able to accurately classify safe/unsafe drivers (AD8, CDT, TMT-A, SMT; sensitivity = 67%, specificity = 94%)
Cox et al. (1998)	29 AD/21 C	AD: 72.0 ± 8.6/C: 70.1 ± 10.0	Simulator	MMSE	AD: 21.2 ± 4.6/C: 28.7 ± 9.6	AD patients more often drove off the road, drove slower, had less brake pressure, and had more difficulty turning left compared to controls
Dawson et al. (2009)	40 AD/115 C	AD: 75.1 ± 7.7/C: 69.4 ± 7.0	On-road	MMSE, CFT, WAIS-R block design, BVRT, TMT, ALVT, JLO, COWA, UFOV, Pelli–Robson, Visual acuity, SFM, Get-up-and-Go	AD: 26.5 ± 2.9/C: not reported	AD patients made more total driving errors compared to controls Lane violations were the most common error
Duchek et al. (1998)	78 AD/58 C	Not reported	On-road	BNT, WMS, BVRT, WFT, WAIS information, bock design, digit symbol, Visual search task, Visual monitoring task, UFOV	–	Error rate and reaction time during visual search were the best predictors of driving performance
Duchek et al. (2003)	50 AD/58 C	AD (mild): 74.2 ± 7.8/AD (very mild): 73.7 ± 7.0/C: 77.0 ± 8.6	On-road	–	–	41% of mild AD and 14% of very mild AD patients were rated as unsafe drivers. Lane changing and signaling were more impaired with increasing dementia severity
Fitten et al. (1995)	13 AD/24 C	AD: 70.0 ± 7.4/C: 71.8 ± 6.8	On-road	MMSE, Clock drawing, Visual tracking, Vigilance, Divided attention, Short-term memory task	AD: 23.2 ± 2.6/C: 29.2 ± 0.9	AD patients drove slower, had lower driving scores and committed more errors than controls
Fox et al. (1997)	19AD/no C	74.3 ± 6.4	On-road	MMSE, JLO, BVRT, TMT, VFDT, WAIS-R picture completion, block design, digit symbol substitution	21.3 ± 2.8	63% of AD patients failed the on-road test Neuropsychological tests were not associated with total driving score
Frittelli et al. (2009)	20 AD/19 C	AD: 72.0 ± 5.5/C: 68.9 ± 6.3	Simulator	MMSE, Visual reaction task	AD: 22.3 ± 3.8/C: 29.1 ± 1.5	AD patients had worse simulated driving performance compared to controls
Hunt et al. (1997)	65 AD/58 C	AD: 73.7 ± 7.8/C: 76.8 ± 8.6	On-road	–	–	29% of AD patients were classified as unsafe drivers versus 3% of controls
Lafont et al. (2010)	20 AD/56 C	AD: 73.3 ± 4.9/C: not reported for total sample	On-road	MMSE, BVRT, Semantic fluency, Cancellation test, DSST, Go/No go test, Stroop, Stop signal, Finger tapping, Reaction time task	AD: 26.4 ± 2.2/C: 29.0 ± 1.1	6/20 (30%) AD patients versus 1/56 (2%) controls were judged unsafe drivers Cognitive functioning (e.g., speed of processing) was associated with an increased risk of unsafe driving (DSST cutoff <25, sensitivity = 75%, specificity = 92%)
Lincoln et al. (2006)	42 AD/33 C	AD: 71.0 ± 8.9/C: 68.5 ± 5.7	On-road	MMSE, SDSA, SORT, Stroop, TEA, VOSP, Letters and Cube, BADS, AMIPB, Balloons test	AD (median): 23/C (median): 29	27% of AD patients were judged as unsafe drivers versus none of the controls Composite battery of cognitive tests was predictive of driving safety (cutoff = 5, sensitivity = 67%, specificity = 100%)
Manning et al. (2014)	75 AD/47 C	AD: 76.7 ± 6.2/C: 71.9 ± 7.8	On-road	MMSE, Clock drawing	AD: 25.1 ± 2.8/C: 29.5 ± 0.7	AD patients had a higher error rate on the road test compared to controls (54.7% versus 14.9%) Clock drawing had low predictive value of driving performance
Ott et al. (2005)	50 AD/no C	75.7 ± 6.6	On-road	–	23.7 ± 4.0	18% of AD patients were classified as unsafe drivers
Ott et al. (2008)	84 AD/128 C	AD: 75.7 ± 7.0/C: 73.5 ± 9.1	On-road	–	AD: 24.1 ± 3.6/C: 29.1 ± 1.1	15% of AD patients failed the on-road test versus none of the controls
Ott et al. (2008)	88 AD/45 C	AD: 75.8 ± 6.9/C: 73.6 ± 9.0	On-road	MMSE, Maze task, CFT, TMT, Finger tapping task, HVLT	AD: 24.0 ± 3.5/C: 29.1 ± 1.1	19% of AD patients were unsafe drivers versus 2% of controls Road navigation was associated with maze navigation Composite battery with maze task, HVLT and TMT-A correctly classified 78.2% of all subjects as safe/unsafe
Paire-Ficout et al. (2016)	18 AD/18 C	AD: 72.7 ± 4.8/C: 74.5 ± 5.4	On-road	MMSE, Verbal fluency, BVRT, Cancellation test, Digit symbol substitution, Go/No go test, Stroop, Stop signal, Finger tapping, Reaction time task, Rotation task	AD: 26.7 ± 1.9/C: 29.3 ± 0.9	AD patients showed planning difficulties during left turns and were slower compared to controls
Piersma et al. (2016)	81 AD/45 C	AD: 72.3 ± 9.4/C: 76.3 ± 4.7	On-road and simulator	MMSE, TMT, Clock drawing, Cube drawing, Maze test, ATAVT, Traffic test, Reaction time, Hazard perception test	AD: 23.2 ± 3.7/C: 28.8 ± 1.1	50.6% of AD patients failed the on-road assessment versus 4.4% of controls AD patients had worse lane keeping on the driving simulator compared to controls
Rizzo et al. (1997)	21 AD/18 C	AD: 71.5 ± 8.5/C: 71.9 ± 5.5	Simulator	RCFT, TMT, WAIS-R block design, WAIS-R information, WAIS-R digit span, BVRT, COWA, Pelli–Robson, UFOV	–	No difference between AD patients and controls in number of crashes Cognitive and visual tests were predictive of crashes
Rizzo et al. (2001)	18 AD/12 C	AD: 73.0 ± 7.0/C: 70.0 ± 4.7	Simulator	RCFT, BVRT, TMT, COWA, WAIS-R block design, WAIS-R information, WAIS-R digit span, Facial Recognition, Pelli–Robson, UFOV	–	Six of 18 AD patients crashed during simulator test versus none of the controls Cognitive tests were predictive of crashes
Stein et al. (2011)	17 AD/63 C	AD (mild): 71.2 ± 8.7/AD (very mild): 74.3 ± 12.2/C: 73.5 ± 6.9	Simulator	–	–	AD patients had impaired vehicle control, difficulties lane keeping, drove slower, and made more judgmental errors compared to controls
Uc et al. (2004)	32 AD/136 C	AD: 75.9 ± 6.2/C: 64.0 ± 11.4	On-road	MMSE, COGSTAT, AVLT, BVRT, RCFT, JLO, WAIS-R block design, TMT part B, COWA, UFOV, SFM, Visual acuity, Contrast sensitivity	AD: 26.3 ± 2.9/C: not reported	AD patients performed worse on a route following task compared to controls Safety errors could be predicted by verbal memory, attention and visuospatial abilities
Uc et al. (2005)	33 AD/137 C	AD: 76.1 ± 6.3/C: 64.3 ± 11.4	On-road	MMSE, COGSTAT, AVLT, BVRT, RCFT, JLO, WAIS-R block design, TMT part B, COWA, UFOV, SFM, Visual acuity, Contrast sensitivity	AD: 26.1 ± 3.0/C: not reported	AD patients identified fewer landmark and traffic signs compared to controls and committed more safety errors
Uc et al. (2006)	61 AD/115 C	AD: 73.5 ± 8.5/C: 69.4 ± 6.7	Simulator	AVLT, RCFT, WAIS-R block design, BVRT, JLO, TMT part B, COWA, COGSTAT, UFOV, Contrast sensitivity, Visual acuity	AD: 25.6 ± 3.8/C: not reported	No differences in crash rates between AD patients and controls AD patients slowed down more abruptly compared to controls
Yamin et al. (2016)	20 AD/21 C	AD: 78.5 ± 7.2/C: 77.0 ± 5.9	Simulator	MMSE, DRS-2, VOSP, TEA, UFOV	AD: 24.0 ± 4.9/C: 29.0 ± 1.3	AD patients performed poorer on almost all driving outcome measures compared to controls
Yi et al. (2015)	28 AD/no C	65.6 ± not reported	Simulator	MMSE, DPT, TMT part B, RFMT	24.1 ± 2.4	AD patients performed best using single, simple auditory driving navigation instructions

*AD* Alzheimer’s disease, *AD8* Assessing Dementia-8 screening interview, *ADL* activities of daily living, *AMIPB* adult memory and information processing battery, *ATAVT* Adaptive Tachistoscopic Traffic Perception Test, *AVLT* Auditory Verbal Learning Test, *BADS* Behavioral Assessment of the Dysexecutive Syndrome, *BNT* Boston Naming Test, *BVRT* Benton Visual Retention Test, *C* controls, *CDR* Clinical Dementia Rating Scale, *CFT* Complex Figure Test, *COGSTAT* composite measure of cognitive impairment, *COWA* Controlled Oral Word Association, *DPT* Doors and People Test, *DRS* Dementia Rating Scale, *DSST* Digit Symbol Substitution Test, *HVLT* Hopkins Verbal Learning Test, *JLO* Judgement of Line Orientation Test, *MMSE* Mini-Mental State Examination, *SBT* Short Blessed Test, *SDSA* Stroke Drivers Screening Assessment, *SFM* Structure from Motion, *SILS* Shipley Institute of Living Scale, *SMT* Snellgrove Maze Test, *SORT* Salford Objective Recognition Test, *TEA* Test of Everyday Attention, *TMT* Trail Making Test, *UFOV* useful field of view, *VOSP* visual object and space perception battery, *WAIS-III* Wechsler Adult Intelligence Scale-III, *WAIS-R* Wechsler Adult Intelligence Scale-Revised, *WCST* Wisconsin Card Sorting Test, *WFT* Word Fluency Test, *WMS* Wechsler Memory Scale

**Table 4:** Types of driving errors categorized by group

Error level	Type of driving error	HD	PD	AD
Tactical				
	Lane changing	X	X	X
	Speed adaptations	X	X	X
	Unsteady car speed	NR	X	X
	Yielding at intersections	NR	X	NR
	Keeping distance	X	X	NR
	Checking blindspot	NR	X	X
	Longer reaction times	X	X	X
Operational				
	Road positioning	X	X	X
	Lane maintenance	X	X	X
	Signaling	NR	X	X
	Steering	NR	X	NR
	Incorrect turning	X	X	X
Strategic				
	Difficulties with road rules	X	X	X
	Inattention while driving	NR	NR	X
	Fewer driving trips	NR	X	X
	Driving less distance	NR	X	NR
	Driving shorter durations	NR	X	X
	Less night time driving	NR	X	NR

Types of driving errors are based on the model by Michon et al. [10]

*X* driving error is reported for this patient group, *NR* not reported in reviewed literature, *AD* Alzheimer’s disease, *HD* Huntington’s disease, *PD* Parkinson’s disease

### Driving and Huntington’s disease

Huntington’s disease (HD) is a hereditary neurodegenerative disorder characterized by choreatic movements, cognitive dysfunction, and psychiatric symptoms [13]. It is caused by a gene mutation located on chromosome 4 [14]. The mean age at onset is between 30 and 50 years, with a mean disease duration of 17–20 years [13]. The earliest cognitive symptoms are characterized by executive dysfunctions, such as difficulties in planning, cognitive inflexibility, and lack of awareness [13, 15]. The cognitive symptoms gradually worsen and eventually result in dementia. Due to the progressive nature of the disease, patients become more dependent in their daily life activities. With the onset of HD during midlife, a lot of patients rely on their car for work and social activities, so patients might find it difficult to decide when to stop driving. However, concern about driving safely is one of the first issues reported by HD patients (33.5%) and has been associated with motor, cognitive, and depressive symptoms [16, 17]. The influence of other psychiatric symptoms, such as aggression and impulsivity, has not yet been investigated.

Only seven studies were found that investigated driving in HD patients [16–22]. Four of these studies used formal driving assessments, either on-road or simulated, to investigate driving competence [18, 20–22]. Due to the limited amount of studies available on HD and driving, the studies that did not investigate driving with formal driving assessments but with questionnaires or retrospective data analyses are also discussed [16, 17, 19]. An observational study investigating the association between different disease aspects of HD with functional changes showed that motor functioning and the Stroop task, measuring cognitive flexibility and information processing, were significantly associated with driving safety [16]. Increased motor impairment was related to a lower likelihood of being able to drive safely as rated by a professional. This study did not include a formal driving assessment. During a semi-structured interview, 11 out of 16 HD participants reported changes in their driving behavior [17]. They reported lower reaction times, had concerns about their safety, and had difficulties multi-tasking. A study that investigated clinical predictors of driving by retrospective patient file reviews showed that cognitive impairment, especially a reduction of psychomotor speed and attention, is a strong risk factor for driving cessation in HD [19]. Increased motor impairments were also associated with not driving a car, but were not a risk factor affecting the decision to cease driving [19]. An early study investigating driving in HD with a driving simulator showed that HD patients committed errors on the operational and tactical level [18]. They were less accurate and had longer reaction times compared to controls [18]. HD patients also had higher error rates in signaling, steering, braking, maintaining speed, and accelerator use. They were more likely to be involved in accidents compared to healthy individuals (58 and 11% respectively) [18]. Still, most of the HD patients in this study continued driving after onset of the disease (53/73). In addition, half of the HD patients that still drive failed an on-road driving assessment [20]. This confirms a limited insight regarding their own driving skills and emphasizes the importance of early evaluation [23–25]. In one study, 14 of the 30 HD patients (47%) failed the on-road driving test [21]. HD patients committed most errors on the operational and tactical levels, including errors in lane positioning, speed adaptations, keeping distance, turning left, and lane changing [21]. They also made more errors in perception of road signs, reflecting errors on the strategic level. Selective attention and disease stage were highly correlated with on-road driving failure in manifest HD [21]. A combination of neuropsychological tasks measuring visual processing speed, visual scanning, and attentional shifting best predicted the pass/fail rate of an on-road driving assessment, instead of a model that also included motor functioning [20]. More recently, it has been reported that some neuropsychological assessments focusing on speed of processing, cognitive flexibility, and visual attentional control seem to be good predictors for driving competence in manifest HD [22].

The results of the reviewed studies showed that driving competence is impaired in patients with HD and that concerns about driving safely are one of the earliest symptoms reported by both patients and families. Especially executive functioning and visuospatial abilities have been related to driving competence in HD. However, due to the limited amount of data, no conclusions can be drawn regarding which cognitive battery is most predictive of driving impairment in HD. None of the studies to date have focused on evaluating driving competency in the earliest stages of HD or in gene mutation carriers without a clinical diagnosis (i.e., premanifest gene carriers), while they often have questions for their physician regarding their driving skills and are most likely in need of a driving evaluation in the near future. Furthermore, no longitudinal studies have been performed investigating driving in HD, so there are no results available about the potential decline in driving competence during the course of the disease. Follow-up measurements are important to determine when driving-related issues become apparent and when to discuss potential driving cessation. It also provides an opportunity to monitor driving from early to more advanced stages of the disease.

### Driving and Parkinson’s and Alzheimer’s disease

Contrary to driving studies in HD, a large number of studies have been performed evaluating driving competence in Parkinson’s disease (PD; *n* = 32) and Alzheimer’s disease (AD; *n* = 31). Three studies compared the driving competence of patients with PD and AD. In the following sections, we will discuss the on-road driving studies first, followed by the studies using driving simulators, and last the studies that also incorporated cognitive functioning in relation to driving performance.

### Parkinson’s disease

Studies using on-road driving assessments (*n* = 22) to evaluate driving competence showed that 12–56% of the PD patients failed an on-road driving test [1, 26–34]. PD patients had a higher number of total driving safety errors compared to control participants. Studies that focused on identifying specific driving errors showed that PD patients are most likely to make errors on a tactical level including difficulties with yielding at intersections [29] and lane changing [1]. They were less likely to check their blind spot, and used their rear view and side mirrors less frequently than controls [1, 35]. Patients with PD also showed a decreased awareness of others, hesitated longer before making a turn, did not accelerate to a proper speed, and were less concentrated [26]. They made more errors in adjusting to different driving situations compared to controls [29] and showed difficulties driving in traffic flow [3]. PD patients made more errors in reversing and car parking [1]. Drivers with PD also had more difficulties with road positioning and driving on roundabouts compared to controls [33]. Most of the errors were present while driving in an urban environment [3]. Errors in the lateral position on the road at low speed and turning left [3] were the best predictors of overall pass/fail driving outcome [32]. Overall, PD patients had an unsteady car speed and tended to drive slower [35–37], especially during distraction [38]. However, it has also been reported that they drove faster on highways compared to controls [37], and had more difficulties adapting their speed at a higher speed [32]. They also identified fewer traffic signs and landmarks compared to controls [39].

On the operational level, PD patients made more incorrect turns and did not signal appropriately compared to controls [26, 35, 36]. They also made more errors in lane maintenance [1, 29, 40]. Strategically, PD patients made fewer driving trips [37, 41], drove less distance, and shorter durations [1, 41] compared to controls. PD patients had a higher preference for driving with a passenger [1, 37], which reported less nighttime driving [29, 37] and more often used alternative transportation [29]. Driving simulator studies (*n* = 12) showed that patients with PD had lower reaction times [42, 43], missed more red lights, and showed impaired accuracy compared to control subjects [42]. Furthermore, they had a higher number of traffic offences [43], more accidents [43, 44], and a worse overall simulator score compared to controls [43]. Patients who passed an on-road driving assessment also performed better on the simulator tests compared to patients who failed the on-road assessment [31]. Patients with PD tended to drive faster than controls and had poorer vehicle control, especially during low contrast visibility conditions [45]. PD patients were found to brake later during incongruent driving conditions [46]. They waited for external cues before they responded, while control subjects initiated a response prior to the cue. This result is similar to another study which found that PD patients relied more on external than internal cues to regulate their driving behavior [47].

A number of studies have incorporated cognitive assessments in an attempt to determine which test performances are associated with the driving competence of patients with PD. Most studies reported an association between cognitive functioning and driving competence [3, 12, 26–28, 31, 32, 36, 38–40, 43, 46, 48–52]. However, some studies also reported no associations between cognition and driving in PD patients [1, 33, 53], so results are inconsistent. Driving errors were particularly associated with lower performances in cognitive flexibility [26, 27, 38, 39, 49, 52], visuoconstructional abilities [26, 36, 39], attention [12, 27, 32, 36, 40, 46], psychomotor speed [46, 51], working memory [12, 49], set shifting [12, 48], information processing [12, 49], contrast sensitivity [27, 31, 43, 48, 51], visual scanning [32], visual acuity [32, 40], speed of visual processing [3, 27, 28, 40], and visual memory [3, 36].

### Alzheimer’s disease

Twenty-three studies were included in this review that investigated driving competence in AD using on-road driving tests. Between 15 and 65% of the AD patients failed an on-road driving assessment [54–64]. They had lower overall driving performance scores compared to controls and committed more overall driving errors [62, 65–71], even in situations that were not considered challenging [54]. Driving performance scores tended to decrease with increasing dementia [57, 63, 72]. The largest decline in driving performance was reported in mild AD patients [57].

On a tactical level, AD patients committed more errors compared to controls in lane positioning [54, 67, 73], lane changing [57, 74], and checking their blind spot [74], and they tended to drive slower [68, 75]. They also had a higher inability to stop the vehicle appropriately [54, 76], and more difficulties avoiding potential collisions compared to controls [76]. Errors in turning [54, 70, 73, 75, 77], signaling [57, 74], and lane maintenance [54, 67, 73] were the most reported errors on the operational level. In contrast, some studies showed no differences between AD patients and healthy individuals in vehicle control [54, 70]. Strategic errors included less attention while driving, slower decision-making, and difficulties with road rules compared to controls [54]. AD patients also had more planning difficulties [75], identified fewer landmarks and traffic signs compared to controls [71], and showed more problems with route following [70].

Comparing driving competence of patients with PD and AD using on-road driving assessments showed that both patient groups committed more overall driving errors compared to controls [73]. These driving errors increased when a concurrent task was included [73]. There are also differences reported between both groups in types of driving errors [74]. Both AD and PD patients committed most errors on the tactical level, but patients with AD also made errors on the operational and strategic levels. Patients with PD committed relatively few operational and strategic errors compared to AD patients [74]. AD patients reported fewer driving trips and drove less miles compared to patients with PD and controls [62, 74]. Contrary, minimal differences between both groups have also been reported [53, 73].

The nine simulator studies reviewed showed that AD patients committed more errors in lane keeping (i.e., more lane deviations) [64, 78–81], turning left [78], and vehicle control [80] compared to controls. AD patients also tended to drive slower [64, 78, 80], took longer to complete the driving tests [78, 79], had less brake pressure [78], and made more judgmental errors (e.g., accidents, collisions) [80]. They failed to stop at traffic lights [80, 81] and exceeded the speed limit more often than controls [81]. Six out of eighteen AD patients crashed during a simulator test [82]. Cognitive and visual tests were predictive of the number of crashes [81–83]. Contrary, no differences in number of crashes between AD patients and controls have also been reported [83]. AD patients performed best when single, simple auditory-only driving navigation instructions were used compared to visual plus audio or visual-only instructions [84].

Drivers with increased cognitive impairments were more likely to be unsafe drivers compared to control subjects [74]. AD patients who failed an on-road assessment performed worse on neuropsychological tasks compared to AD patients who passed the on-road test [64]. Decreased performances on cognitive tests measuring speed of processing [62, 67, 73, 85], executive functioning [56, 74], attention [56, 70–72, 76], memory [67, 68, 70, 71, 73, 76], set shifting [62, 71, 73], visuoconstructional and visuospatial abilities [56, 67, 70, 71, 73, 74, 76], visual searching [56, 67, 72], and visual tracking [68] have been associated with worse scores on driving outcome variables and increased error rates in patients with AD. A composite battery of tests was more predictive of driving than individual tests [60, 67], and cognitive performance was more predictive of driving ability than AD diagnosis alone [85]. However, no correlations between neuropsychological outcome measures and on-road evaluations have also been reported [58, 77].

### Self-assessment of driving performances

In addition to differences in driving performances, there are also differences reported in the evaluation of driving ability performed by patients, caregivers, and physicians. One study reported that PD patients rated their own driving performances lower than controls [50]. Contrary results showed that about 20% of the PD and AD patients misjudged their own driving ability [3, 43]. In addition, the rating performed by a neurologist (*M* = 8.0) was more optimistic compared to the rating performed by a driving instructor (*M* = 5.1) and psychologist (*M* = 5.7) [3]. Spouses tended to overestimate the driving ability of AD patients [86]. Ratings performed by an adult child were more related to driving outcome variables than ratings performed by spouses [86]. Self-ratings of driving ability performed by AD patients and ratings by spouses were significantly higher than ratings by an independent evaluator and physician [65, 87]. Ratings by a clinician were poorly associated with an on-road driving test, but not with naturalistic driving [86]. However, these clinician ratings were still more associated with driving performance compared to the self-evaluation by patients and the evaluation by spouses [65]. Caregivers did acknowledge general problems with driving, but still rated the AD patients driving competence significantly higher than an independent rater [87].

### Driving simulator use

Since on-road driving assessments in patients with neurodegenerative disorders might be unsafe, an alternative is to evaluate driving competence with a simulator. Driving simulators provide the opportunity to present challenging situations and events in a standardized setting, with a high reproducibility compared to on-road driving assessments where situations cannot be manipulated [88]. Simulators are also used to train novice drivers before they start their on-road driving lessons [89]. Results of a concurrent and discriminant validity study comparing an on-road driving assessment with driving simulator tasks revealed that a driving simulator is a valid measure of driving performance for research purposes [90]. The driving simulator outcomes were able to discriminate between drivers with different levels of experience. In a study with elderly drivers, over 65% of the variability in the on-road assessments could be explained by driving simulator outcomes [91]. Adding a driving simulator increased the total variance explained by a potential screening battery to 60 and 94% [31, 43], suggesting that a driving simulator might be a useful screening tool to evaluate driving fitness. Studies that described the use of simulators for rehabilitation and training purposes in various disorders showed promising results, with more patients passing an on-road assessment after training with a simulator [92]. The lower ecological validity of a simulator, however, could be a disadvantage, because participants may prefer driving a real vehicle. It is also important to keep in mind that a reduction of driving performance measured with a simulator might reflect the adaption to the simulator itself and not actual driving ability. Therefore, it is necessary to further investigate the differences between disease groups and healthy individuals to minimize the effects of simulator use. In addition, the relationship between on-road performances and simulator driving should be further explored to determine whether simulator outcome measures are, indeed, consistent with on-road driving performance.

A common issue in simulator research is the existence of simulator sickness, which is comparable to motion sickness [93, 94]. It includes dizziness, nausea, vomiting, and sweating. The symptoms of simulator sickness are typically less severe than motion sickness and tend to decrease with multiple exposure and time [94, 95]. Dropouts in simulator studies have been related to simulator sickness, with up to one-third of the participants experiencing signs of simulator sickness [64, 84, 91]. The duration and configuration of the driving scenario influence this dropout rate [96]. For example, scenarios including more turns and sudden stops increase the risk for simulator sickness. Older age, female gender, and prior history of motion sickness have also been associated with higher susceptibility of experiencing simulator sickness [97, 98]. However, dropouts are not necessarily those subjects with the poorest performances [98, 99]. Several theories have been proposed to explain the occurrence of simulator sickness [94]. A conflict between structures within the sensory and vestibular systems has been the most widely excepted theory [94, 100]. When using a simulator to evaluate driving competence, this side-effect should be taken into consideration by excluding patients who experience simulator sickness or by screening beforehand. However, this might result in selection bias that should be accounted for. For more information regarding the topic of simulator sickness, we refer to the systematic review by Classen et al. [97].

## Discussion

The majority of studies investigated driving competence of patients with a neurodegenerative disorder with on-road driving assessments, and this is considered the gold standard. Results showed worse driving performances in patients compared to controls, although there is a large variability in types of driving errors. Most errors are committed in lane changing, lane maintenance, lower reaction times, and larger variabilities in speed. Inconsistencies in results might be attributable to different methods and outcome measures. In addition, there is a large heterogeneity in the patient population and sample sizes (range *n* = 16–266). Specific types of driving errors are often not investigated and only global pass/fail ratings are reported. For research purposes, it is important to determine which types of driving errors are most common and if these errors also pose a safety hazard for the patient and environment. Some errors might be manageable and do not necessarily mean that the patient should cease driving. For example, errors on the strategic level, such as difficulties with planning a route, are less dangerous and more manageable than errors concerning reacting to other road users and vehicle control. Adaptations to the vehicle might also increase the time that a patient is still able to drive safely. PD patients were better drivers when they used an automatic car compared to a manually operated car [34]. Driving simulators have the potential to assist in investigating driving competence, but there are still limited results available. In addition, there is the phenomenon of simulator sickness that should be considered when using a simulator [97]. There is also variability in types of driving simulators (i.e., manufacturers) and scenarios that are used. Driving simulator studies often use motorway scenarios, because they are less susceptible to simulator sickness. These scenarios are useful to investigate reaction times and speed adaptations, but might not properly reflect the driving ability on the road in an environment with more distractors. Driving scenarios including rural or urban areas, with more traffic, different speeding zones, and sudden events, might be more difficult due to the higher demand on cognitive functioning. The utility of a driving simulator to predict on-road driving behavior in both research and clinical practice has to be further explored.

In most studies, more than half of the patients with a neurodegenerative disorder were classified as safe drivers. This suggests that a majority of the patients can still drive safely. Therefore, professionals should not base their recommendations about potential driving cessation solely on the presence of a clinical diagnosis [85]. Individual evaluations are important and changes in driving performance should be monitored regularly, preferable every year. Due to the progressive nature of neurodegenerative disorders, formal retesting of driving skills is recommended even if the driver license has been renewed for an extended period of time. Although this is not a review on driving competence in the normal elderly population, the influence of aging should be taken into consideration. However, the mean ages in the reviewed studies were relatively young (HD = 43.1 years, PD = 66.4 years, AD = 74.0 years), and most analyses were corrected for the effects of age. This suggests that older age alone is not a criterion to continue or cease driving.

Overall, the findings reported in the reviewed studies suggest that cognitive functioning is associated with safely operating a vehicle. The current literature suggests some consensus on which cognitive domains are associated with decreased driving competence. Diminished functioning in the visuocontructional, visuospatial, executive, and attentional domains has consistently been associated with impaired driving. Specific neuropsychological assessments are partially predictive of driving outcomes, but there is currently no valid screening battery that can accurately be used in the clinical practice. There are limited cut-off scores available, so it is still difficult to translate performances on neuropsychological tests to clinical recommendations. The most promising screening batteries, with sensitivity and specificity ranging between 61 and 94%, included the Trail Making Test (TMT), useful field of view (UFOV), Pelli–Robson, and Symbol Digit Modalities Test (SDMT). Baseline and follow-up assessments are necessary to further validate the usefulness of these tests. Recently, it has been reported that a combination of assessments (i.e., clinical interviews, neuropsychological assessments, and driving simulator outcomes) best predicted fitness to drive in patients with AD [64]. Furthermore, composite neuropsychological test batteries have been more predictive of driving performances than separate tests [26, 31, 32, 40, 50, 60, 67]. This suggests that a composite battery including multiple cognitive domains might be a reliable predictor of driving performance. However, this approach should be further validated before the practical application of such a screening battery can be determined.

Our review showed that there is still a gap in the current driving literature. Only a limited amount of longitudinal studies have been performed in AD and PD but none in HD. Follow-up is important for early intervention and to monitor changes over time. Moreover, there is a large discrepancy in the amount of studies available regarding driving in HD compared to PD and AD. Since the etiology of HD is known, this disorder could potentially be a good prototype to investigate changes in driving competency and the association with cognitive decline. Furthermore, there is the opportunity to investigate both symptomatic and asymptomatic gene carriers in an attempt to detect at which point in the disease driving-related issues become apparent. This is particularly useful for the clinical practice and to establish guidelines for patients, families, and caregivers. An important factor differentiating HD from PD and AD is the age at onset. HD typically occurs during midlife with a mean age at onset between 30 and 50 years, while signs and symptoms of PD and AD are most often developed later in life [13, 101, 102]. With this relatively young age at onset of HD, most patients still rely on their car for employment and social activities. Therefore, discussing driving ability is important at an early stage of the disease. Furthermore, no studies have been performed regarding the association between psychiatric symptoms (e.g., irritability and apathy) and driving. These are important signs of HD that can already be present at early stages of the disease and might influence driving behavior [15].

Both HD and PD can be distinguished from AD by the presence of motor disturbances, but the nature of cognitive deficits also differs. The cognitive impairments observed in AD can be considered a cortical dementia, whereas HD and PD are mainly characterized by subcortical changes [103, 104]. In HD and PD, problems in the executive domain are most commonly observed, while in AD, memory impairments are more pronounced [105, 106]. This different expression of cognitive profiles might also affect driving in distinctive ways. In addition, specific subtypes of motor signs in PD (i.e., tremor versus dyskinesia) potentially influence the ability to operate a car. Differences between these specific subtypes in fitness to drive have not been studied to date. However, it has been reported that patients with postural instability and gait disorder PD subtype failed an on-road driving assessment more often than patients with the tremor dominant subtype of PD (46 versus 7%) [32]. Different motor subtypes can also be distinguished in HD (chorea versus hypokinesia-rigidity) and these subtypes have been associated with between different cognitive profiles [107, 108]. These differences in symptomatology should be further investigated in relation with driving performance to increase knowledge about important individual differences.

An important issue to keep in mind is the limited insight of patients with neurodegenerative disorders into their own disabilities. We believe that it is important to discuss driving in the outpatient clinic in the presence of spouses or relatives to ascertain a more objective point of view. However, some partners might find it difficult to express their concerns with the patient there. The role of the physicians is important to start the discussion at the right time and to provide the necessary referrals. On the same note, it is interesting to further explore the patient’s perspective regarding driving cessation, since some studies did report that there are patients who modify their driving behavior [109, 110].

In general, there are numerous difficulties in performing driving research in neurodegenerative disorders that should be considered when developing study protocols. An important issue is the presence of potential selection bias. Patients might fear that their license will be revoked and, therefore, do not want to participate in driving-related studies [111]. Patients who are less confident about their driving ability might be less willing to participate. In addition, there are safety concerns when evaluating driving performances. Other issues are the relatively small sample sizes, lack of control groups, and differences in methodology.

## Conclusion

Based on the current available literature, it is not possible to draw one final conclusion if and when patients with neurodegenerative disorders should be restricted in their driving. Driving requires optimal cognitive functioning and lower performances on neuropsychological assessments might serve as a first indicator of driving incompetence. However, there is currently no validated screening battery available. Some patients with neurodegenerative disorders are still able to drive safely, so a restriction of driving solely based on a clinical diagnosis is unwarranted. None of the studies to date have resulted in practical guidelines that can be implemented in clinical settings. We are of the opinion that formal retesting should be mandatory due to the progressive nature of neurodegenerative diseases. Longitudinal studies are, therefore, necessary to determine when driving-related issues become apparent and to investigate the progression rate of driving incompetence. Further studies focusing on establishing specific evidence-based guidelines that take differences between disorders into consideration are needed. The lack of patient insight into their own driving competence should be further explored and emphasizes the need to quantify driving status.
